# Geometric Self-Calibration of YaoGan-13 Images Using Multiple Overlapping Images

**DOI:** 10.3390/s19102367

**Published:** 2019-05-23

**Authors:** Guo Zhang, Mingjun Deng, Chenglin Cai, Ruishan Zhao

**Affiliations:** 1 State Key Laboratory of Information Engineering in Surveying, Mapping and Remote Sensing, Wuhan University, Wuhan 430079, China; guozhang@whu.edu.cn; 2 School of Information Engineering, Xiangtan University, Xiangtan 411000, China; chengcailin@126.com; 3 School of Geomatics, Liaoning Technical University, Fuxin 123000, China; zhaoruishan333@163.com

**Keywords:** YaoGan-13, geometric accuracy, self-calibration

## Abstract

Geometric calibration is an important means of improving the absolute positioning accuracy of space-borne synthetic aperture radar imagery. The conventional calibration method is based on a calibration field, which is simple and convenient, but requires a great deal of manpower and material resources to obtain ground control points. Although newer cross-calibration methods do not require ground control points, calibration accuracy still depends on a periodically updated reference image. Accordingly, this study proposes a geometric self-calibration method based on the positioning consistency constraint of conjugate image points to provide rapid and accurate calibration of the YaoGan-13 satellite. The proposed method can accurately calibrate geometric parameters without requiring ground control points or high-precision reference images. To verify the absolute positioning accuracy obtained using the proposed self-calibration method, YaoGan-13 Stripmap images of multiple regions were collected and evaluated. The results indicate that high-accuracy absolute positioning can be achieved with a plane accuracy of 3.83 m or better for Stripmap data, without regarding elevation error. Compared to the conventional calibration method using high-accuracy control data, the difference between the two methods is only about 2.53 m, less than the 3-m resolution of the image, verifying the effectiveness of the proposed self-calibration method.

## 1. Introduction

The Chinese YaoGan-13 (YG-13) satellite mission, launched in November 2015, is equipped with a high-resolution synthetic aperture radar (SAR) X-band sensor. Synthetic aperture radar image products can be acquired using ScanSAR, Stripmap, and Sliding-spot modes, with the last of these providing SAR images at a very high resolution of about 0.5 m. The launch of YG-13 provided China with the ability to acquire high-resolution SAR images globally [1,2]. However, despite the strong capability of YG-13 in acquiring high-resolution SAR images, most of the images obtained by the satellite have exhibited poor absolute positioning accuracy, due to systematic timing offsets in the SAR system, including the time shift between the radar time and Global Positioning System (GPS) time (i.e., azimuth or along-track), and the internal electronic delay of the SAR instrument itself (i.e., the range delay time) [3]. As a result, the application of YG-13 images in activities such as resource monitoring has been significantly restricted.

Using high-accuracy control data, a conventional geometric calibration method can eliminate systematic errors, such as those experienced by YG-13 (including the internal electronic delay of the instrument and systematic azimuth shifts), improving the geometric positioning accuracy of the images. The conventional geometric calibration method has been thoroughly studied by many researchers and fully validated using the ERS-1/2, ENVISAT-ASAR, ALOS-PALSAR, TerraSAR-X/TanDEM-X, Sentinel-1A/1B, YaoGan-13, GaoFen-3, and other high-resolution satellites [4,5,6,7,8,9,10,11,12,13,14]. However, the conventional calibration method requires satellites to acquire images of calibration fields prior to conducting geometric calibration, reducing its timeliness in practical applications. Additionally, the conventional calibration method typically uses a high-precision corner reflector to generate control data, which can be expensive.

In the optical remote sensing field, many scholars have researched methods for geometric self-calibration that do not rely on control data, achieving notable success [15,16]. In the field of SAR geometric calibration, researchers have also begun to study geometric calibration without field calibration control data. Deng et al. performed cross calibration without using corner reflectors or high-precision digital elevation models to improve the absolute positioning accuracy of images collected by the GaoFen-3 (GF-3) satellite [2]. However, this method does not completely eliminate dependence on control data, as it still requires high-precision reference images. To the authors’ knowledge, no studies of completely independent SAR geometric self-calibration have been reported to date.

In this paper, a novel self-calibration method is proposed to determine the systematic timing offsets in the SAR system, independent of ground control points (GCPs). This method uses at least three images containing overlapping areas and takes advantage of the spatial intersection residual between conjugate points in these images to detect the timing offsets. The proposed method is free from the constraint of field control data present in traditional calibration methods. To demonstrate the accuracy of the proposed method, a series of experiments using Stripmap images collected by YG-13 is presented. The results show that the proposed method is able to effectively eliminate the systematic errors, due to the internal electronic delay of the instrument and the systematic azimuth shifts. After calibration, the plane absolute positioning accuracy of the YaoGan-13 Stripmap image was better than 3.83 m, just larger than the 3-m resolution of the images, verifying the effectiveness of the proposed method.

## 2. Methodology

### 2.1. Fundamental Theory of the Proposed Method

**Figure 1:**
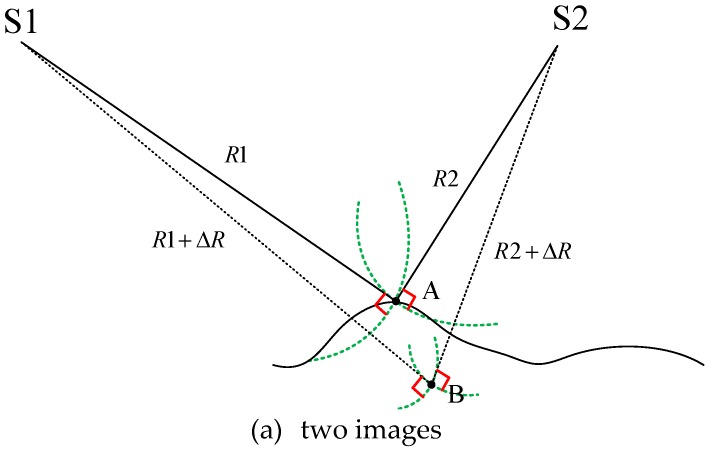

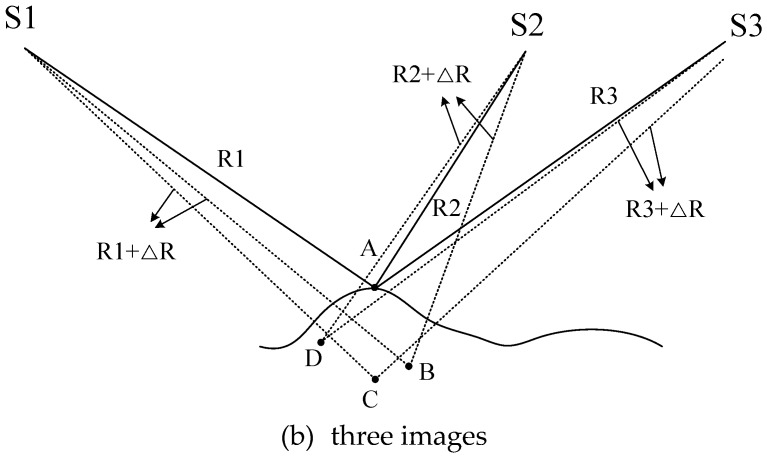
Schematic diagram of synthetic aperture radar (SAR) image geometric self-calibration.

Figure 1 illustrates the proposed method of SAR image geometric self-calibration. In Figure 1a, S1 and S2 correspond to the two SAR antenna phase centers when the ground surface at Point A is photographed twice. The slant range between S1 and A is R1 and the slant range between S2 and A is R2, and so the intersection point between R1 and R2 is Point A. If a slant range measurement error ΔR exists due to the geolocation parameter error, the slant range R1 becomes R1 + ΔR, the slant range R2 becomes R2 + ΔR, and the new spatial intersection is at Point B. In this way, the spatial intersection point is affected by the error of the geometric positioning parameters. However, this could be true for another case: The changes in the slant range R1 and R2 can be caused by the position error of the ground point, because the ground surface position of Point A is unknown. Therefore, we can conclude that when using only two images, the exact reason for the changes in slant ranges R1 and R2 is uncertain.

Accordingly, in Figure 1b, we add a third image, and the slant range between S3 and A is given by R3. However, due to the aforementioned errors, slant range R3 becomes R3 + ΔR. New spatial intersection points then exist between S3 and S1 at Point C, and between S3 and S2 at Point D. If the change in slant range is caused by a ground point error, then spatial intersection Points B, C, and D should all be the same. If, however, the change in slant range is caused by a geolocation parameter error, then the spatial intersection Points B, C, and D are likely to be different. This difference is called the spatial intersection residual. A minimum spatial intersection residual can thus be used as a constraint condition for solving the self-calibration equation.

### 2.2. Proposed Geometric Self-Calibration Method

The physical imaging process of SAR can be represented by the slant range equation and Doppler equation [17,18].

The slant range equation is given by:

(1) $$R=\sqrt{{\left(X_{s}-X\right)}^{2}+{\left(Y_{s}-Y\right)}^{2}+{\left(Z_{s}-Z\right)}^{2}},$$

where *R* is the slant range between the sensor and the target, and $\left(X_{s},Y_{s},Z_{s}\right)$ and (X,Y,Z) are the sensor and target position vectors, respectively.

The Doppler equation is given by:

(2) $$f_{D}=-\frac{2}{\lambda R}\left[\left(X_{s}-X\right)X_{v}+\left(Y_{s}-Y\right)Y_{v}+\left(Z_{s}-Z\right)Z_{v}\right],$$

where $f_{D}$ is the Doppler center frequency for the SAR image, λ is the SAR wavelength, and $X_{v},Y_{v},Z_{v}$ is the phase center velocity vector of the SAR antenna.

Based on the slant range and Doppler equations, the geometric self-calibration model of a SAR image can be written as follows:

(3) $$\left\{ \begin{array}{l} R=\sqrt{{\left(X_{s}-X\right)}^{2}+{\left(Y_{s}-Y\right)}^{2}+{\left(Z_{s}-Z\right)}^{2}}+R_{s}+R_{atmo}\\ f_{D}=-\frac{2}{\lambda (R-R_{s}-R_{atmo})}\left[\left(X_{s}-X\right)X_{v}+\left(Y_{s}-Y\right)Y_{v}+\left(Z_{s}-Z\right)Z_{v}\right] \end{array}\right.,$$

where $R_{s}$ is the slant range correction and $R_{atmo}$ is the atmospheric path delay, calculated using the atmospheric delay correction model [19,20,21]. 

The error equations for Equation (3) are given by:

(4) $$\left\{ \begin{array}{l} V_{R}=\frac{\partial R}{\partial X}\Delta X+\frac{\partial R}{\partial Y}\Delta Y+\frac{\partial R}{\partial Z}\Delta Z+\frac{\partial R}{\partial R_{s}}\Delta R_{s}+\left(R\right)-R\\ V_{f_{D}}=\frac{\partial f_{D}}{\partial X}\Delta X+\frac{\partial f_{D}}{\partial Y}\Delta Y+\frac{\partial f_{D}}{\partial Z}\Delta Z+\frac{\partial f_{D}}{\partial R_{s}}\Delta R_{s}+\left(f_{D}\right)-f_{D} \end{array}\right.,$$

where (R) and $\left(f_{D}\right)$ are the approximate values of the slant range and Doppler center frequency, respectively.

Equation (4) can be expressed as the following matrix equation:

(5) V=AK−L,

where

$K={\left[\Delta X,\Delta Y,\Delta Z,\Delta R_{s}\right]}^{T}$$V={\left[V_{R},V_{f_{D}}\right]}^{T}$,
$L={\left[l_{R},l_{f_{D}}\right]}^{T}={\left[R-(R),f_{D}-(f_{D})\right]}^{T}$, and
$A={\left[\begin{array}{l} a_{11},a_{12},a_{13},a_{14}\\ a_{21},a_{22},a_{23},a_{24} \end{array}\right]}^{T}$.

The values of the partial derivatives in Equation (4) are:

$a_{11}=\frac{X-X_{s}}{\sqrt{{\left(X-X_{s}\right)}^{2}+{\left(Y-Y_{s}\right)}^{2}+{\left(Z-Z_{s}\right)}^{2}}}$,
$a_{12}=\frac{Y-Y_{s}}{\sqrt{{\left(X-X_{s}\right)}^{2}+{\left(Y-Y_{s}\right)}^{2}+{\left(Z-Z_{s}\right)}^{2}}}$,
$a_{13}=\frac{Z-Z_{s}}{\sqrt{{\left(X-X_{s}\right)}^{2}+{\left(Y-Y_{s}\right)}^{2}+{\left(Z-Z_{s}\right)}^{2}}}$,
$a_{14}=1$,
$a_{21}=\frac{2X_{v}}{\left(R-R_{s}-R_{atmo}\right)\lambda }$,
$a_{22}=\frac{2Y_{v}}{\left(R-R_{s}-R_{atmo}\right)\lambda }$,
$a_{23}=\frac{2Z_{v}}{\left(R-R_{s}-R_{atmo}\right)\lambda }$, and
$a_{24}=\frac{2\left[\left(X_{s}-X\right)X_{v}+\left(Y_{s}-Y\right)Y_{v}+\left(Z_{s}-Z\right)Z_{v}\right]}{\lambda \left(R-R_{s}-R_{atmo}\right)\left(R-R_{s}-R_{atmo}\right)}$,

where the initial value $R_{s}=0$ and the initial values of [X Y Z] are calculated using the least squares spatial point intersection. 

According to the least squares principle of indirect adjustment, the normal form of Equation (5) can be expressed as follows:

(6) $$A^{T}AK=A^{T}L.$$

The expression of the solution of Equation (6) can then be obtained by:

(7) $$K={\left(A^{T}A\right)}^{-1}A^{T}L.$$

The corrected ground coordinates of conjugate point [ΔX ΔY ΔZ] and the slant range error $dR_{s}$ can then be obtained, using an iterative procedure to calculate ${\left[\Delta X,\Delta Y,\Delta Z,\Delta R_{s}\right]}^{T}$, expressed by the following algorithm steps:

1. Select the conjugate points. Measure the image plane coordinates of the conjugate points on each image;
2. Obtain information on geometric positioning parameters. Find and calculate the imaging time t, Doppler center frequency $f_{D}$, and slant range R of the target point from the auxiliary files. Find and calculate the position vector $X_{s},Y_{s},Z_{s}$ and velocity vector $X_{v},Y_{v},Z_{v}$ of the satellite;
3. Determine the initial value of the unknown parameters. The measurement errors of the slant range and the systematic azimuth shifts are typically not large, so the initial values of the slant range correction and systematic azimuth shifts can be set to 0. The initial values of [X Y Z] are then calculated using a least squares spatial point intersection [22];
4. Calculate the approximate values of the slant range and the Doppler center frequency for each conjugate point. The approximate values of these unknown parameters are then substituted into the slant range equation (Equation (1)) and the Doppler equation (Equation (2)) to calculate the approximate values of the slant range (R) and the Doppler center frequency $\left(f_{D}\right)$, respectively, for each conjugate point;
5. Calculate the coefficient and constant terms of the error equation (Equation (4)), point by point to establish the error equation;
6. Calculate the coefficient matrix $A^{T}A$ and constant term $A^{T}L$ of the normal equation (Equation (6)) to establish the normal equation;
7. Calculate the slant range and ground coordinate corrections of the conjugate points and add them to the corresponding approximate values to obtain the new approximate ground coordinates of the conjugate points and the slant range correction;
8. Calculate the slant range error. Check if the calculation converges by comparing the ground coordinate corrections of the conjugate points and the slant range error with the prescribed error limits: The correction of the slant range error is usually evaluated against a limit of 0.1 m. When the correction of the slant range error is less than 0.1 m, the iteration ends and proceeds to Step 9, otherwise, repeat Steps 4–8 with the newest approximation until the error limits are met;
9. Calculate the systematic azimuth shifts $\Delta t_{a}$. Calculate the image plane coordinates of the conjugate points by using the inverse location algorithm with the new approximate ground coordinates of the conjugate points, then update the azimuth imaging time [23]. Recalculate the position vectors $X_{s},Y_{s},Z_{s}$ and the velocity vectors $X_{v},Y_{v},Z_{v}$ of the satellite. Set the slant range correction to 0 and the initial ground coordinates of the conjugate points used are the results of the previous iteration. Repeat Steps 4–9 until the correction of the systematic azimuth shift is less than the limit;
10. The accurate values of ${\left[\Delta X,\Delta Y,\Delta Z,\Delta R_{s}\right]}^{T}$ and $\Delta t_{a}$ are obtained.

## 3. Experiment and Analysis

### 3.1. Experimental Study Areas and Data Sources

**Figure 2:**
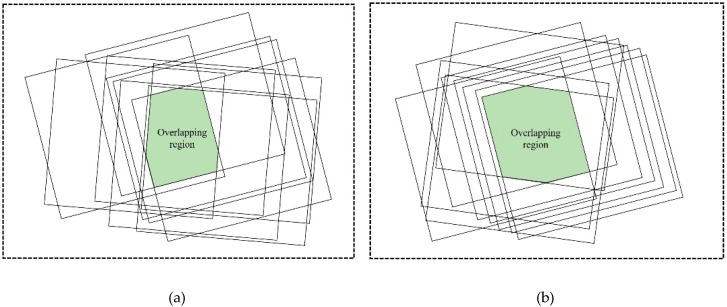
Spatial distribution of experimental data in (**a**) Calibration Group A (200 MHz and 24.4 μs), and (**b**) Calibration Group B (150 MHz and 24.4 μs).

In order to verify the accuracy of the self-calibration method proposed in this paper, Stripmap images from the YaoGan-13 satellite acquired between 18 December, 2015 (2015-12-18) and 30 March, 2016 (2016-03-30) were used as experimental data. The resolution of the Stripmap images was 3 m and the swath width was 10 km. The internal electronic delay of the instrument (slant range error) is related to the bandwidth and pulse width of the radar signal. Therefore, the experimental data were divided into two groups according to the differences in the bandwidth and pulse width: Calibration Group A, with a bandwidth of 200 MHz and a pulse width of 24.4 μs, summarized in Table 1, and Calibration Group B, with a bandwidth of 150 MHz and a pulse width of 24.4 μs, summarized in Table 2. As can be seen in Figure 2, the data in Calibration Group A and Calibration Group B cover overlapping areas. The conjugate points were selected in this overlapping area.

### 3.2. Results of Proposed Self-Calibration Method

According to the theory presented in Section 2.1, the proposed geometric self-calibration method requires at least three images; these necessary redundant observations can be obtained by adding calibration images to the data set. As shown in Table 3 (for Calibration Group A) and Table 4 (for Calibration Group B), the number of images was increased sequentially according to the image acquisition time, resulting in a total of eight image combinations in Calibration Group A and eight image combinations in Calibration Group B. Three pairs of well-distributed conjugate points were then manually acquired from the calibration images. Using these conjugate points, self-calibration was conducted using the proposed method. The slant range correction and systematic azimuth shifts obtained by self-calibration using each image combination in Calibration Groups A and B are also shown in Table 3 and Table 4, respectively, in which it can be seen that the geometric calibration parameters obtained using different calibration combinations are similar. The difference between the maximum and minimum value of the slant range correction was 1.83 m, while the difference between the maximum and minimum value of the systematic azimuth shifts was about 0.153 ms with an accompanying 1.16-m azimuth geolocation error, given a spacecraft velocity of 7600 m/s. These ranges demonstrate that the calibration results are stable.

### 3.3. Validation of Self-Calibration Accuracy

**Figure 3:**
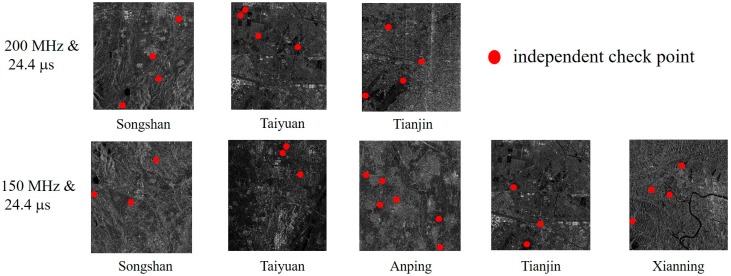
Distribution of independent check points (ICPs) in the validation images.

**Figure 4:**
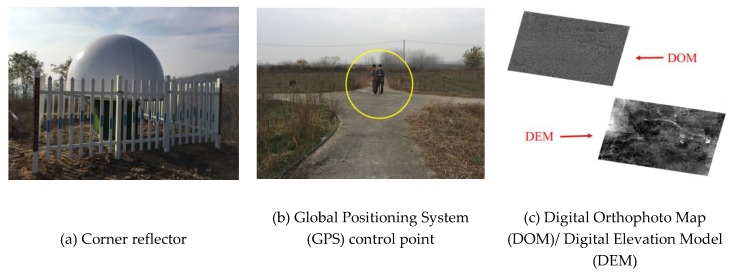
Sources of the three types of control data used for verification.

In order to verify the accuracy of the proposed method for self-calibration, Validation Groups A and B, consisting of Stripmap images collected from the Songshan, Taiyuan, Anping, Xianning, and Tianjin areas, were evaluated with bandwidth and pulse width parameters matching Calibration Groups A and B and control data, as provided in Table 5. The terrain of the Taiyuan, Tianjin, and Anping test sites was almost flat, while that of Songshan and Xianning test sites was hilly. Several independent check points (ICPs) were manually extracted from the control data and the distributions of these ICPs in the validation images are shown in Figure 3. The sources of the three types of control data used are shown in Figure 4. The difference between the predicted location of the ICPs in the object space and their measured locations is the absolute positioning error, generally expressed in the north, east, and plane dimensions, separately. In this system, the “Plane” error is numerically equal to the square root of the sum of the squares of the “East” and “North” errors.

The root-mean-square error (RMSE) of the absolute positioning accuracy (north, east, and plane) was then calculated for the images in Validation Group A (Table 6) and Validation Group B (Table 7) before and after calibration. In the tables, Plan 1 represents the absolute positioning accuracy without calibration, while Plans 2–9 represent the absolute positioning accuracy for the different self-calibration combinations defined in each group. The results of the statistical analysis show that without calibration, the absolute positioning accuracy of both Validation Group A and Validation Group B is very poor at 29.47 m and 22.76 m, respectively. After calibration, the absolute positioning accuracy clearly shows significant improvement: High-accuracy absolute positioning is achieved with a plane accuracy of 3.83 m or better for Validation Group A and 3.41 m or better for Validation Group B. 

To further illustrate the effectiveness of the proposed self-calibration method, corner reflectors were used as control points to conduct a conventional field calibration for Calibration Groups A and B. The absolute positioning accuracies of Validation Groups A and B using the reflectors were then compared with those determined by the self-calibration method, as shown in Table 8. The maximum positioning errors from among Plans 2–8 (shaded) in Table 6 and Table 7 were selected to represent the self-calibration results. Notably, the difference between the positioning accuracy provided by the proposed self-calibration method and the conventional field calibration is not large: For Validation Groups A and B, there was a difference of 2.53 m and 1.74 m, respectively, in plane positioning accuracy, both smaller than the 3-m resolution of the images. 

## 4. Conclusions

It is generally known that the most critical aspect of improving the geolocation accuracy of satellite imagery is geometric calibration. Conventional field calibration and cross calibration methods cannot satisfy the demands for fast and accurate calibration. In this study, a novel self-calibration method based on the positioning consistency constraint of conjugate points was proposed to calibrate satellite geometric parameters without requiring ground control points or high-precision reference images. The proposed method uses at least three overlapping images and takes advantage of the spatial intersection residual between corresponding points in the images to calculate systematic errors (such as the internal electronic delay of the instrument and systematic azimuth shifts). YaoGan-13 Stripmap-mode images were collected as experimental data and analyzed using the proposed method. The results show that the plane absolute positioning accuracy after self-calibration is better than 3.83 m. The difference in accuracy compared to the conventional method was only about 2.53 m, which is less than the 3-m resolution of the images, verifying the effectiveness of the proposed self-calibration method.

## Figures and Tables

**Table 1:** Calibration Group A image data.

Bandwidth and Pulse Width	Image ID	Imaging Time	Central Angle	Orbit	Look-Side
200 MHz and 24.4 μs	A1	2015-12-28	36.1°	Asc	R
	B1	2016-01-03	43.1°	Desc	R
	C1	2016-01-16	26.8°	Asc	R
	D1	2016-01-18	31.4°	Desc	R
	E1	2016-01-19	37.5°	Asc	L
	F1	2016-03-03b	24.8°	Asc	L
	G1	2016-03-11	37.1°	Desc	R
	H1	2016-03-26a	23.6°	Desc	R
	I1	2016-03-27a	15.2°	Desc	L
	J1	2016-03-27b	31.2°	Asc	L

Desc = Descending; Asc = Ascending; L = Left; R = Right.

**Table 2:** Calibration Group B image data.

Bandwidth and Pulse Width	Image ID	Imaging Time	Central Angle	Orbit	Look-Side
150 MHz and 24.4 μs	A2	2015-12-29	46.1°	Desc	R
	B2	2016-01-04	46.9°	Asc	L
	C2	2016-01-07a	54.6°	Desc	R
	D2	2016-01-17a	50.9°	Desc	R
	E2	2016-01-17b	48.9°	Asc	R
	F2	2016-03-10	45.5°	Asc	R
	G2	2016-03-15	48.0°	Asc	R
	H2	2016-03-26b	50.4°	Asc	L
	I2	2016-03-29b	38.0°	Asc	R
	J2	2016-03-30	53.8°	Asc	R

Desc = Descending; Asc = Ascending; L = Left; R = Right.

**Table 3:** Geometric calibration results for Calibration Group A (200 MHz and 24.4 μs).

Combination	Slant Range Correction (m)	Systematic Azimuth Shifts (ms)
A1-B1-C1	15.96	−0.126
A1-B1-C1-D1	15.72	−0.131
A1-B1-C1-D1-E1	15.88	−0.140
A1-B1-C1-D1-E1-F1	16.35	−0.126
A1-B1-C1-D1-E1-F1-G1	16.19	−0.118
A1-B1-C1-D1-E1-F1-G1-H1	17.55	−0.120
A1-B1-C1-D1-E1-F1-G1-H1-I1	16.61	−0.128
A1-B1-C1-D1-E1-F1-G1-H1-I1-J1	16.57	−0.134

**Table 4:** Geometric calibration results for Calibration Group B (150 MHz and 24.4 μs).

Combination	Slant Range Correction (m)	Systematic Azimuth Shifts (ms)
A2-B2-C2	17.34	0.009
A2-B2-C2-D2	17.16	−0.000
A2-B2-C2-D2-E2	16.90	−0.083
A2-B2-C2-D2-E2-F2	15.81	−0.131
A2-B2-C2-D2-E2-F2-G2	15.73	−0.133
A2-B2-C2-D2-E2-F2-G2-H2	16.12	−0.130
A2-B2-C2-D2-E2-F2-G2-H2-I2	17.13	−0.144
A2-B2-C2-D2-E2-F2-G2-H2-I2-J2	16.97	−0.137

**Table 5:** Image data used in Validation Groups A and B.

Validation Group	Bandwidth and Pulse Width	Test Site	Imaging Time	Control Data	Number of ICPs
A	200 MHz and 24.4 μs	Songshan	2016-03-29b	Six Corner reflectors	4
		Taiyuan	2016-05-28	1:5000 DOM/DEM	4
		Tianjin	2016-05-29	1:2000 DOM/DEM	4
B	150 MHz and 24.4 μs	Songshan	2016-04-02	Six Corner reflectors	3
		Taiyuan	2016-06-01	1:5000 DOM/DEM	3
		Anping	2016-06-09	GPS control points	6
		Tianjin	2016-06-10	1:2000 DOM/DEM	3
		Xianning	2016-06-12	GPS control points	4

**Table 6:** Comparison of absolute positioning accuracy of Validation Group A before and after compensating for geometric calibration parameters.

Calibration Plan	Combination	Absolute Positioning Accuracy (m)		
		North	East	Plane
Plan 1 *	None	6.93	28.64	29.47
Plan 2	A1-B1-C1	1.65	3.06	3.47
Plan 3	A1-B1-C1-D1	1.74	3.41	3.83
Plan 4	A1-B1-C1-D1-E1	1.70	3.16	3.59
Plan 5	A1-B1-C1-D1-E1-F1	1.52	2.46	2.89
Plan 6	A1-B1-C1-D1-E1-F1-G1	1.56	2.70	3.12
Plan 7	A1-B1-C1-D1-E1-F1-G1-H1	1.12	0.87	1.42
Plan 8	A1-B1-C1-D1-E1-F1-G1-H1-I1	1.43	2.06	2.51
Plan 9	A1-B1-C1-D1-E1-F1-G1-H1-I1-J1	1.45	2.11	2.56

* Plan 1 denotes no calibration performed.

**Table 7:** Comparison of absolute positioning accuracy of Validation Group B before and after compensating for geometric calibration parameters.

Calibration Plan	Combination	Absolute Positioning Accuracy (m)		
		North	East	Plane
Plan 1 *	None	5.41	22.11	22.76
Plan 2	A2-B2-C2	1.44	1.54	2.11
Plan 3	A2-B2-C2-D2	1.40	1.71	2.21
Plan 4	A2-B2-C2-D2-E2	0.96	1.97	2.19
Plan 5	A2-B2-C2-D2-E2-F2	0.94	3.18	3.31
Plan 6	A2-B2-C2-D2-E2-F2-G2	0.95	3.27	3.41
Plan 7	A2-B2-C2-D2-E2-F2-G2-H2	0.89	2.82	2.96
Plan 8	A2-B2-C2-D2-E2-F2-G2-H2-I2	0.70	1.75	1.89
Plan 9	A2-B2-C2-D2-E2-F2-G2-H2-I2-J2	0.74	1.91	2.05

* Plan 1 denotes no calibration performed.

**Table 8:** Comparison between conventional field calibration and proposed self-calibration method.

Validation Group	Calibration Method	Absolute Positioning Accuracy (m)		
		North	East	Plane
A	Conventional field calibration	1.04	0.78	1.30
	Self-calibration	1.74	3.41	3.83
	Difference between the two methods	—		2.53
B	Conventional field calibration	0.70	1.52	1.67
	Self-calibration	0.95	3.27	3.41
	Difference between the two methods	—		1.74
